# The anesthesiologist’s guide to swine trauma physiology research: a report of two decades of experience from the experimental traumatology laboratory

**DOI:** 10.1007/s00068-024-02542-7

**Published:** 2024-05-23

**Authors:** Mattias Renberg, Tomas Karlsson, Albin Dahlquist, Claire Luckhurst, Jenny Gustavsson, Ulf Arborelius, Mårten Risling, Mattias Günther

**Affiliations:** 1 Department of Clinical Science and Education Södersjukhuset, Section of Anesthesiology and Intensive care, Stockholm, Sweden; 2 Department of Neuroscience, Section of Experimental Traumatology, Karolinska Institutet, Biomedicum– 8B, SE-171 77 Stockholm, Sweden

**Keywords:** Swine physiology research, Experimental traumatology, Anesthesiology, Porcine research, Trauma

## Abstract

**Purpose:**

Swine are one of the major animal species used in translational research, with unique advantages given the similar anatomic and physiologic characteristics as man, but the investigator needs to be familiar with important differences. This article targets clinical anesthesiologists who are proficient in human monitoring. We summarize our experience during the last two decades, with the aim to facilitate for clinical and non-clinical researchers to improve in porcine research.

**Methods:**

This was a retrospective review of 337 swine with a mean (SD) weight 60 (4.2) kg at the Experimental Traumatology laboratory at Södersjukhuset (Stockholm south general hospital) between 2003 and 2023, including laboratory parameters and six CT-angiography examinations.

**Results:**

Swine may be ventilated through the snout using a size 2 neonatal mask. Intubate using a 35 cm miller laryngoscope and an intubating introducer. Swine are prone to alveolar atelectasis and often require alveolar recruitment. Insert PA-catheters through a cut-down technique in the internal jugular vein, and catheters in arteries and veins using combined cut-down and Seldinger techniques. Cardiopulmonary resuscitation is possible and lateral chest compressions are most effective. Swine are prone to lethal ventricular arrhythmias, which may be reversed by defibrillation. Most vital parameters are similar to man, with the exception of a higher core temperature, higher buffer bases and increased coagulation. Anesthesia methods are similar to man, but swine require five times the dose of ketamine.

**Conclusion:**

Swine share anatomical and physiological features with man, which allows for seamless utilization of clinical monitoring equipment, medication, and physiological considerations.

**Supplementary Information:**

The online version contains supplementary material available at 10.1007/s00068-024-02542-7.

## Introduction

Swine are one of the major animal species used in translational research, surgical models, and procedural training and are increasingly used as the choice of nonrodent species in preclinical toxicologic testing of pharmaceuticals. There are unique advantages to the use of swine in laboratory models given that they share with man similar anatomic and physiologic characteristics involving the cardiovascular, urinary, integumentary, and digestive systems [1, 2]. However, the investigator needs to be familiar with important anatomic, histopathologic, and clinicopathologic features of the swine, and needs to consider specific differences. Many excellent and comprehensive textbooks [1, 2] and review articles [3–5] describe and discuss these different aspects. However, there are no specific sources of information for anesthesiologists. Clinical anesthesiologists are trained in advanced human catheter insertions and monitoring. Ultrasound guided procedures are now standard practice, and patient monitoring is becoming more advanced, and at the same time less invasive. For example, pulse contour analysis algorithms estimate cardiac output from peripheral arterial lines, and transcutaneous blood oxygenation measurements may outdate blood-gas analysis in the future. Still, physiology research in swine models must rely on direct measurements. Algorithms validated on man may be inaccurate in swine and are not disclosed by manufacturers due to intellectual property limitations. Therefore, invasive monitoring including pulmonary-arterial catheters are still standard experimental practice. Swine of human weight share many anatomical features with man, but there are prominent differences to acknowledge. In this article, we summarize our experience during the past two decades, with the aim to facilitate for clinical anesthesiologists to improve in human sized porcine physiology research. We focus on the pre-experimental anesthesia and instrumentation and discuss common features and misconceptions.

## Materials and methods

This is a retrospective study summarizing our experience, in publications listed here: [6–25]. All trials were short, terminal, and terminated within 12 h. All studies were approved by and conducted in accordance with ethics approvals for animal research, issued by the Swedish Board of Agriculture (approval numbers A89-04, A 17 − 08, A 80 − 07, S22-13, S3-15, 1470, 12,578 − 2020, 2021 − 01181). The experiments were performed under veterinary supervision between 2003 and 2023, and 337 animals were included in this analysis. The swine were specific-pathogen-free (SPF), Yorkshire/Swedish land race crossbred race and were housed in an accredited animal facility for 3–5 days prior to the experiment and fed a standard diet with free access to tap water. The ambient room temperature was maintained at 21–22 °C with 12-hour light/12-hour darkness cycles. The swine weighed a mean (SD) 60 (4.2) kg and were approximately 4 months old. Sexual maturity is reached by both the male and female swine at about 5–6 months of age [26]. All males were castrated. Data are available in the electronic notebook at Karolinska Institutet upon reasonable request. Statistical analyses were performed using GraphPad Prism v. 10.1.2. Six CT-angiography examinations [27] were analyzed by clinical radiologists at Södersjukhuset. New 3D renderings were done using syngo.via (Siemens Healthineers) and Sectra PACS (Sectra medical).

## Results

### Anatomical considerations

**Fig. 1:**
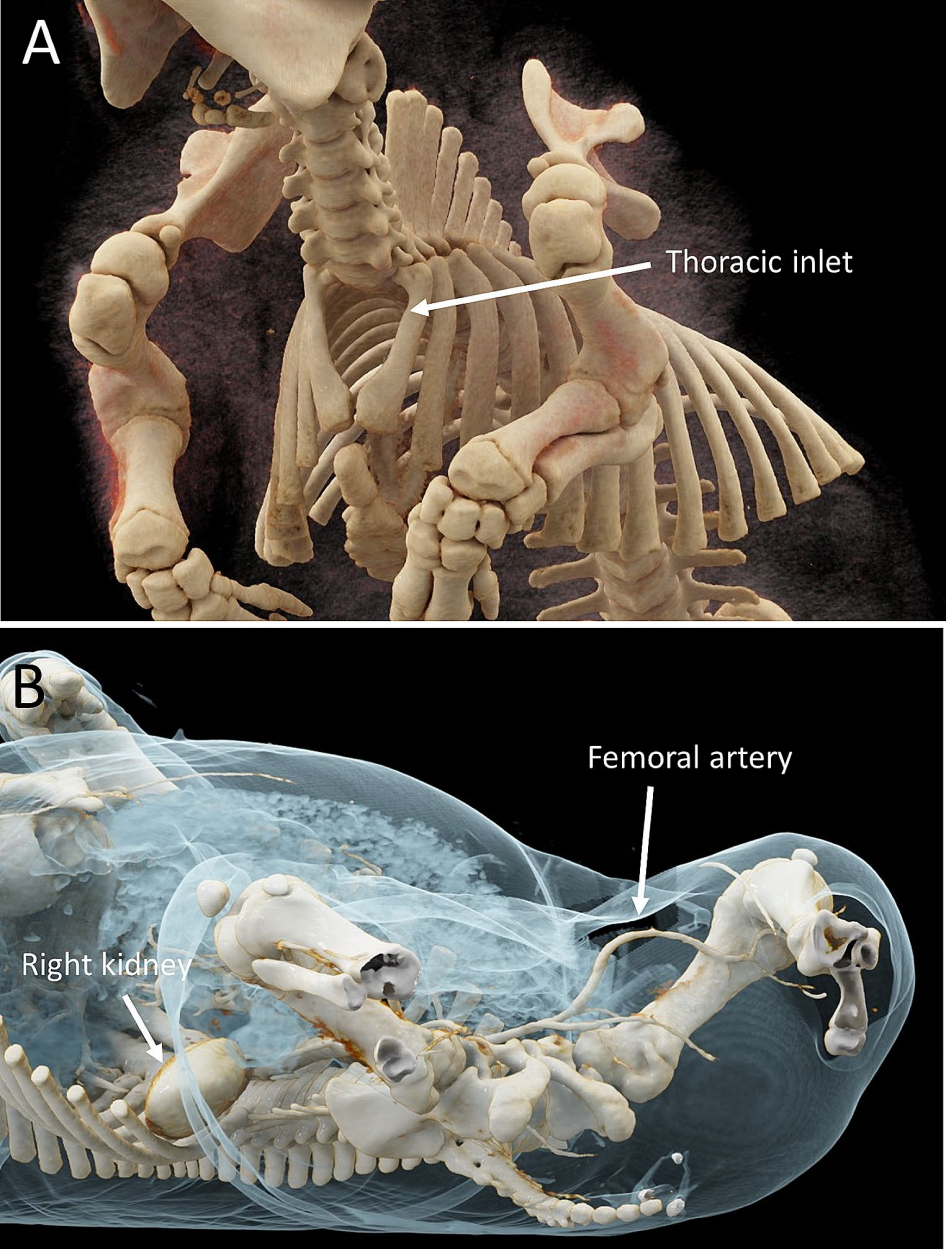
3D renderings of computed tomography showing (A) the configuration of the neck and thoracic cage. (B) femoral artery exiting the pelvis and point of access in the hind leg

**Fig. 2:**
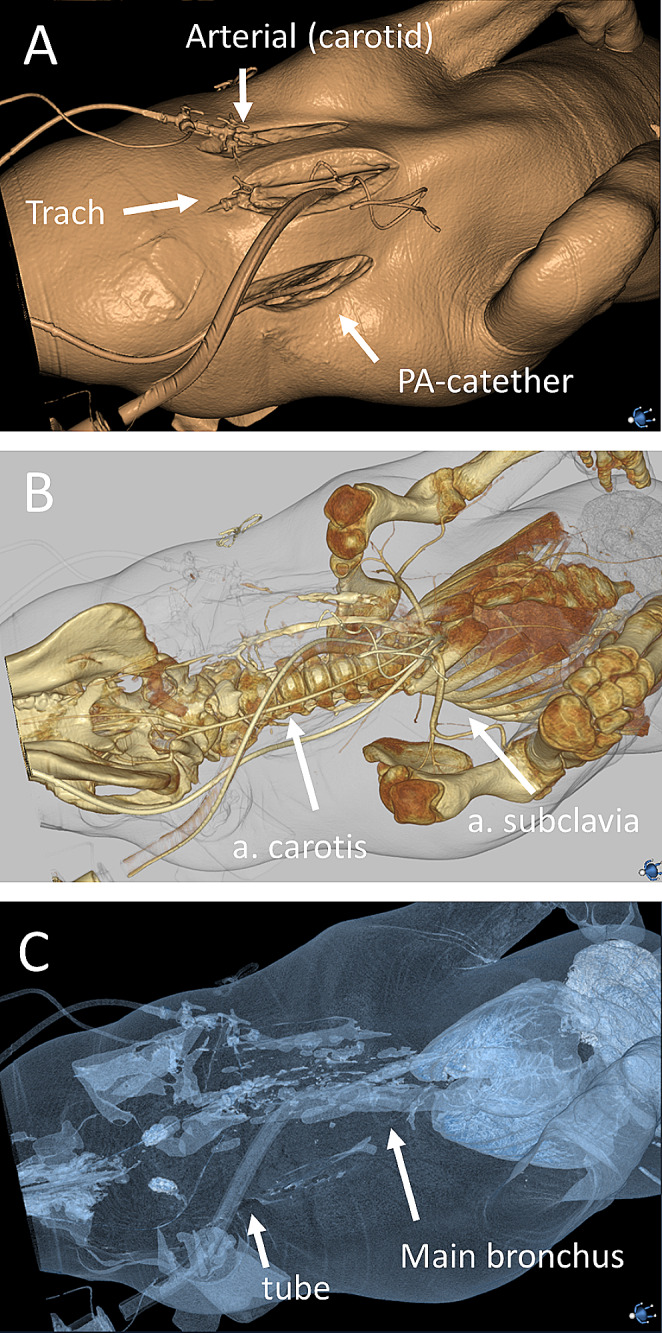
3D renderings of computed tomography showing vascular and ventilatory accesses. (A) The PA-catheter is placed on the right side of the trachea by cut-down technique. Tracheostomy (or intubation) in the trachea. An arterial line may be placed in the left internal carotid, if not placed in the foreleg

The swine thorax is more conical in shape than in man, with a greater difference in width between the first pair of ribs compared to the lower pairs in combination with an elevated sternum. Swine lack clavicles. The first pair of ribs form the thoracic inlet with a significantly smaller cross-sectional area than in man (Fig. 1A). The aortic arch has two branches as opposed to three in man: the brachiocephalic artery and the left subclavian artery. The brachiocephalic artery dividing into the right subclavian artery and a bicarotid trunk which in turn divides into the common carotids. The aorta trifurcates distally into the external iliac arteries and an internal iliac trunk, which in turn branches into the internal iliac arteries and a caudal artery. The external iliac artery divides into deep and superficial femoral artery while still within the pelvis. The latter follows a path similar to the human femoral artery with a short, fairly superficially located inguinal segment accessible for puncture when extending the hindleg of the swine (Fig. 1B). The external jugular vein is the most accessible vein for puncture as the internal jugular vein is located deeply in the cervical soft tissue and of smaller diameter. The internal and external jugular veins both merge with the axillary veins bilaterally. A left azygous (hemiazygos) vein drains the intercostal veins and enters the coronary sinus of the heart. There is an intrathoracic segment of the inferior vena cava caudal to the entrance of the right atrium which is entirely surrounded by lung parenchyma (accessory lobe of the right lung). This differs from man where the heart abuts the diaphragm. The small intestine is mainly located to the right in the abdomen. It is potentially very mobile as the mesenteric attachment to the dorsal peritoneum is less extensive than in man. The ascending colon, mainly located to the left, is coiled forming a proximal outer and a distal inner helix. 3D renderings of computed tomography show vascular and ventilatory accesses (Fig. 2A-C). The PA-catheter is placed on the right side of the trachea by cut-down technique. Tracheostomy (or intubation) in the trachea. An arterial line may be placed in the left internal carotid, if not placed in the foreleg.

### Airway management and ventilation

**Fig. 3:**
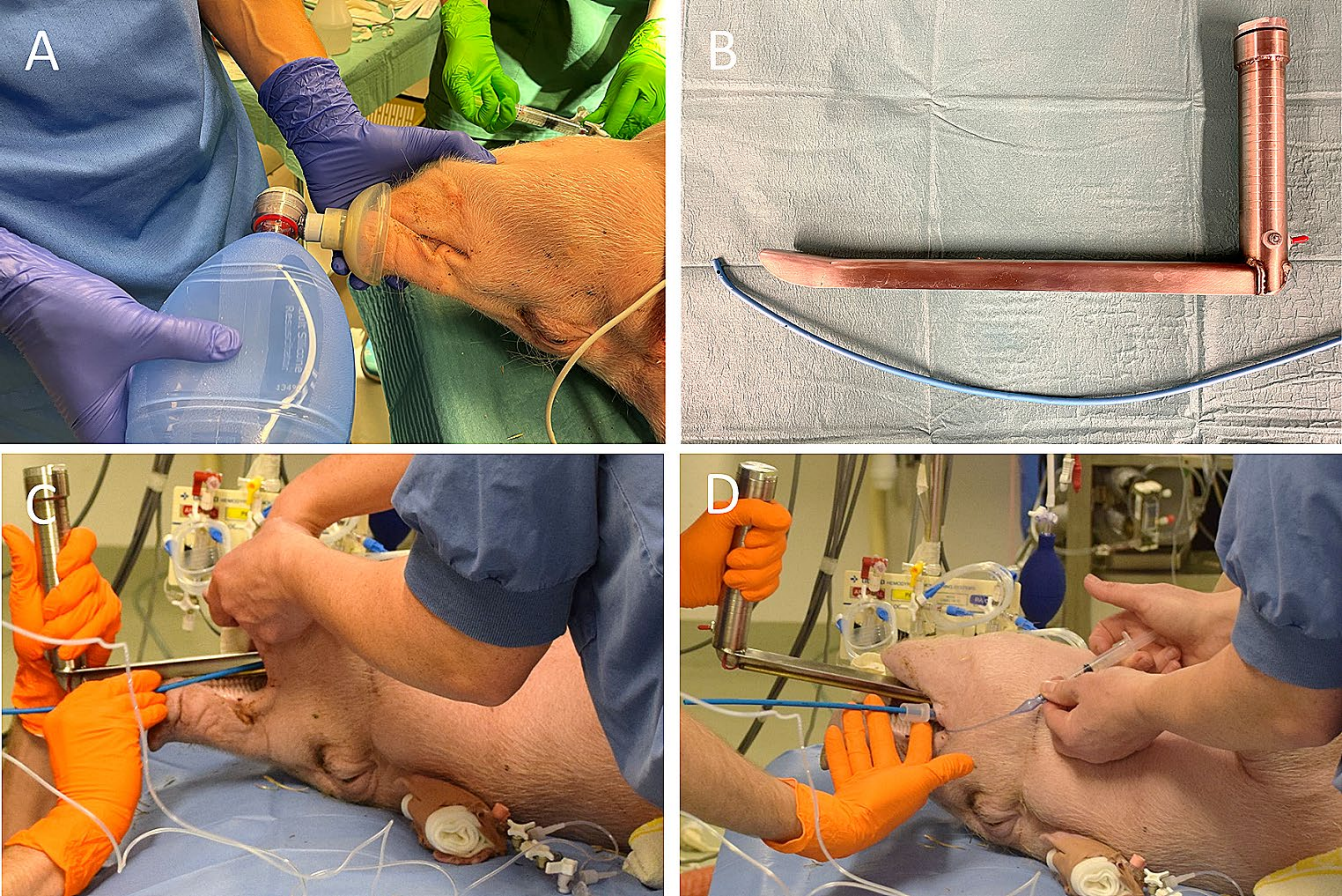
Airway management. (A) Ventilation is easily achieved using a size 2 neonatal mask over the nostrils and keeping the mouth shut. (B) A custom-made 35 cm miller-type laryngoscope and an intubating introducer (bougie catheter) are used for intubation. (C) Intubation is performed by visualizing the larynx with the miller laryngoscope and placing the bougie catheter in the trachea, on which the tube is passed. An assistant is holding the lower jaw (D) The tube is inserted all the way to the corner of the mouth and the cuff is inflated

Physiology research in swine commonly require advanced airway management with either endotracheal intubation or tracheostomy [28]. Successful handling of the airway requires skills, practice, and anatomical knowledge of the swine as the oropharyngeal anatomy may be challenging. The swine has a long, thick tongue and a long, narrow oropharyngeal cavity with an elongated soft palate. Above the esophagus, the pharyngeal diverticulum protrudes from the wall. The introitus of the larynx can be covered by a long epiglottis, in the dorsal position and has a blunt angle in relation to the lateral ventricles which may complicate intubation [29], with the risk of hypoxia and death [30]. In our experience, most adverse events can be avoided. We preoxygenate the swine using a neonatal mask (size 2), which fits perfectly on the snout (Fig. 3A). Although other reports state that swine ventilation is “almost impossible” [31], we find it is easy to ventilate the swine through the snout, while keeping the mouth shut. This technique also allows for non-invasive ventilation (NIV). In our experience, without preoxygenation, saturation will decrease faster than healthy, equally weighted humans, when intubating in apnea. We suggest a ventro-dorsal position and not dorsoventral for orotracheal intubation. This provides for a smooth and fast airway [30]. We find that endotracheal intubation is facilitated by using an initial 30-degree angle of the head, when introducing the laryngoscope and subsequently re-angulating to -10 degrees approaching the larynx, in relation to the bed. This optimizes the line of vision in relation to the laryngeal axis. Pulling the tongue to the side while advancing a Miller-style laryngeal blade (blade length 35 cm, height 2 cm, width 2.5 cm, handle height 18 cm) further facilitates the procedure. Elevation of the epiglottis with the blade enhances the laryngeal vision. Due to the long distance from the mouth to the larynx, using an intubating introducer (bougie catheter) facilitates the intubation procedure (Fig. 3B). A resistance may arise a few centimeters after the larynx aperture because of the obtuse angle arising from the lateral ventricles. This is bypassed by a 180 degrees downward rotation of the tip of the bougie. The resistance may also occur when advancing the endotracheal tube over the bougie caused by the shape of the bevel and is again solved with a 90-180-degrees rotation of the tube. By taking these measures, we have close to 100% success rate. If snout ventilation or endotracheal intubation is not possible after the induction of anesthesia, it may be necessary to perform an emergency front-of-neck airway, which we have investigated with clinically active anesthesiologists as operators [22]. This is best performed using a surgical cricothyroidotomy technique, through a midline vertical skin incision, dissection down to cricothyroid membrane and thereafter a horizontal incision and a 90-degree rotation to enable bougie insertion. While maintaining the patency of the hole with the scalpel, a bougie is introduced over which a size 6 endotracheal tube is slid down [22, 32] (Fig. 3C-D). After intubation, swine are prone to atelectasis formation [33]. Although this is also a well-known phenomenon in man [34], in our experience it is more severe in swine than in healthy humans in supine position, particularly when a protocol with zero end-expiratory pressure (ZEEP) is desired. We perform alveolar recruitment maneuvers liberally after intubation. Supine versus prone position does not seem to affect the aerated lung volume, neither with ZEEP nor PEEP, or after lung recruitment [35].

### Peripheral and central venous catheters

**Fig. 4:**
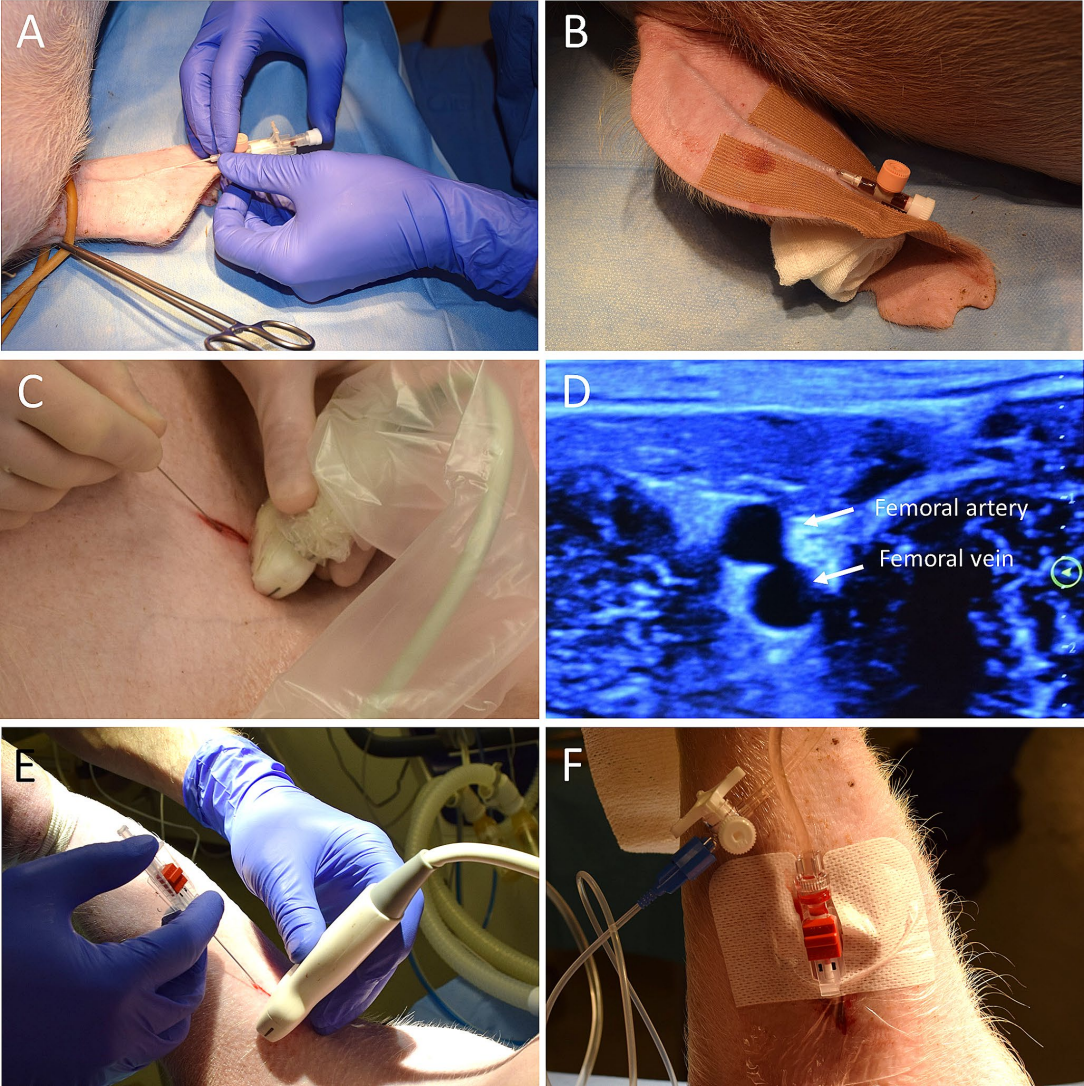
Vascular access. (A) The easiest venous access is obtained in the ear. (B) To keep the access open, place a rolled-up gauze in the ear to prevent the ear from folding. (C) Needle puncture for Seldinger technique access of the femoral artery through a skin incision perpendicular to the ultrasound probe. (D) Out of plane ultrasonogram of the femoral artery and femoral vein (scale in centimeters). (E) arterial needle is best placed in the foreleg, by first making a small incision and protruding the needle using ultrasound. (F) Keep the foreleg extended by traction to keep the arterial line open

Peripheral venous access is easiest obtained via the auricular vein. To keep the access open, we place a rolled-up gauze in the ear to prevent the ear from folding (Fig. 4A-B). If the auricular vein does not work, it is also possible to find peripheral veins in the foreleg or hindleg, although veins in these positions are more obscure than in man. In our experience, central venous access is technically more challenging than in man. Swine internal jugular veins may be too small and too deep for ultrasound guided Seldinger technique, although this has been described [36–38]. Historically, farm swine have been restrained with a hog snare and blood obtained by blind puncture from the anterior vena cava [39]. We have standardized a procedure of surgical access, which is reliable and less time consuming. A 10 cm skin incision is made on the right side of the trachea. Finger dissection is done, and exposure of the vein is performed with blunt forceps. This is a fast, easy-to-learn technique with low risk of complications, albeit more invasive than Seldinger technique. A central venous line or PA-catheter may then be inserted and sutured to the vein. It should be noted that it is also possible to access the jugular vein with percutaneous technique, and the techniques may be used equivalently.

### PA-catheter

A PA-catheter is still the best option to achieve direct measurements of CO, left ventricle preload (wedge pressure) and SvO_2_. PA-catheters function reliably in large swine but require some considerations. We insert the PA-catheters in the right internal jugular vein after a cut-down as described above. The length of the PA catheter from the insertion point at the internal jugular vein to the right ventricle was mean (range) 22 (10) cm (*n* = 53), and the length to PA-pressure was mean (range) 37 (11) cm (*n* = 51) and the length to wedge pressure was mean (range) 41 (11) cm (*n* = 69). PA-catheters carry an increased risk of arrhythmias, primarily ventricular tachycardia when measuring wedge pressures [40, 41]. In our experience, swine risk lethal ventricular arrythmias to a higher degree than man, and not only in relation with wedge pressure readings. Lethal arrhythmias can occur at any time and leads to immediate circulatory collapse. The condition is reversible if treated immediately and does usually not cause any metabolic derangements which require termination of the experiment. If a ventricular tachycardia or signs of impending ventricular tachycardia such as sudden onset of frequent ventricular extrasystoles occur and intervention is acceptable with respect to the research protocol, our standard approach is to retract the PA-catheter 5–10 cm and defibrillate. The PA-catheter can then be re-inserted once the situation has stabilized. In our experience, adrenaline is best given earlier then in the algorithms for man but in a lower dose (0.1 mg). We aim to create as little circulatory distortion as possible, as it may interfere with the experimental protocol. 1 mg of adrenaline may be excessive and unnecessary. An action list in case of circulatory collapse due to primary arrythmia is shown in Table 1.

**Table 1:** Action list in case of ventricular fibrillation

1. Retract PA catheter 5–10 cm.
2. Adrenaline 0.1 mg IV.
3. Start the defibrillator.
4. Set amperage to 250 J (biphasic).
5. Charge the defibrillator.
6. Defibrillate with spatulas close to the thorax on both sides of the sternum.
7. Unless sinus rhythm and return of spontaneous circulation (ROSC), start cardiac compressions at a rate of 100 per minute.
8. Repeat defibrillation after 2 min.
9. Adrenaline 0.1 mg IV.

### Arterial access

Swine skin is structurally similar to human epidermal thickness and dermal–epidermal thickness ratios [4], and arterial access through standard technique have been described in porcine models [36, 37, 42]. A small incision through the epidermis may facilitate when placing an arterial line, with or without Seldinger technique, because the skin is thicker than in man, and precision is lost when penetrating the thicker skin of the swine. This is a matter of experience, and affluent swine-experimentalists may omit the skin incision. The difference compared to literature may be due to different weights, ages, and races of the swine. We use a combined cut-down and Seldinger technique to ensure easy introduction of dilators and to reduce the risk of catheter bending. After identification of the best spot for vascular access with both in- and out-of-plane ultrasound technique, a 1–2 cm perpendicular incision is made through the skin and the fascia (Fig. 4C-D). Vascular access is established with Seldinger technique, using the smallest diameter needle as possible, and without a syringe attached. This gives immediate feedback, reduces needle movement in the vessel and does not cause any significant blood flow. In our experience, this is not associated with more complications than in man, and we have placed 7 French introducers for REBOA in the femoral artery with more than 90% first attempt and 100% second attempt success rates. Due to the short access length of the femoral artery before it disappears from ultrasonic view in the pelvis, we extend the hindlimb, to ensure that the artery is straight and keep the access point as distal as possible, to optimize for a potential second attempt proximal to the first location.

When placing a peripheral arterial catheter in the brachial artery, keeping the forelimb moderately extended during the monitoring further reduces the risk of movements and bending and keeps the anatomy more favorable for the arterial access. Ultrasound guided penetration with a standard arterial line is normally sufficient (Fig. 4E-F). We use techniques that increase first attempt success. Identify a proximal location for a second attempt before the first attempt.

### Urinal catheters

Urethra catheterization is impossible in male swine due to penile anatomy [1, 2]. It is possible to surgically insert a urinal catheter with a pouch suture. We routinely insert a 10 French suprapubic catheter by ultrasound guidance. Identification of bladder location using an intramuscular needle and a standard syringe for urine aspiration is also possible. The bladder is easily localized if filled, but it may also be empty or positioned behind the large intestines and therefore not identifiable by ultrasound. In these cases, the normal procedure is to wait until the bladder fills and becomes visible for ultrasound guided insertion.

### Cardiopulmonary resuscitation (CPR)

CPR is possible in swine. The elevated sternum and ribcage differ from man. Chest compressions are effective when compressing from the top down in supine position, but it may be more effective to place the swine on the side and compress laterally. Defibrillations are done by placing electrodes on each side of the protruding sternum. Less power is needed to defibrillate due to the short distance between the electrodes and relatively less air compared to man, which gives lower impedance.

### Coagulation

Swine are hypercoagulable compared to man, as shown by us [17] and others [43–46]. This may cause clotting of venous and arterial lines to a higher extent than in man. In our experience, heparin coating or heparin in arterial catheters are not necessary if the arterial access is flushed with crystalloids using a standard 5–10 mL syringe after every blood sample and when the arterial wave form show signs of dampening. Avoid all forms of bending of catheters as this will cause clotting. A 1 mL/min infusion of crystalloid of will decrease the risk of clotting. When hemorrhaging from an arterial line (for example the femoral artery), the risk of clotting increases if the blood flow is below 12 mL/min. We do not experience clotting if the blood flow is above 12 mL/min, and heparin or citrate is normally not required.

### Vital parameters and blood chemistry

Vital parameters and blood chemistry are presented in Table 2. All values are collected at baseline, after induction of anesthesia and before initiation of the experiment. This may confound normal ranges. However, the intention was to collect parameters in a realistic experimental environment. Heart rates were < 100 beats/minute. The heart rate is normally much higher in young than in adult swine [4]. Swine are hyperthermic compared to man [47]. Hb was lower compared to man [44, 48–50]. Platelets were increased compared to man [44, 48–50]. Fibrinogen was within reference values of man but lower compared to earlier studies [44, 46, 51]. D-dimer was within reference values of man [44]. Activated partial thromboplastin time (APTT) increased compared to man, in contrast to earlier studies [46, 51]. We and others previously showed that rotational thromboelastometry (ROTEM) had faster clotting time, steeper clot angle and greater clot strength compared to man [17, 45, 46]. Prohormone of brain natriuretic peptide (pro-BNP), c-reactive protein (CRP), cortisol, adrenaline, and neuron specific enolase (NSE) were not possible to analyze reliably in swine. Markers of inflammation in swine reported to work are serum amyloid-A (SAA), haptoglobin (HP) and pig major acute phase protein (Pig-MAP) [52].

**Table 2:** Baseline values

	*n*	Mean; SD	Median	IQR (25; 75)	Range (min-max)
Weight (kg)	337	60 ± 4.2	60	57; 63	48–72
Temperature (°C)	315	38.4 ± 0.77	38.4	37.9; 38.9	36-40.3
Heart rate (beats/min)	318	97.8 ± 20	93.8	83.1; 110	60.9–188
SAP (mmHg)	92	131 ± 19.4	132	118; 147	89–166
MAP (mmHg)	316	112 ± 19	110	99.9; 124	57.1–169
CVP (mmHg)	302	3.96 ± 4.35	3.62	0.75: 7.08	-8.0-16.3
MPAP (mmHg)	288	20.9 ± 6.7	20	16.3;24.9	6.0-41.7
Wedge pressure (mmHg)	49	5.2 ± 3.5	4.9	2.4;6.7	0.2–18.5
SvO_2_ (%)	298	71.1 ± 10.8	73	66.7; 78	26-91.5
CCO (l/min)	299	5.62 ± 1.31	5.55	4.7; 6.5	2.33–9.7
Respiratory rate (breaths/min)	279	27.2 ± 15.3	20	16; 37	8-71.5
Minute ventilation (l/min)	280	8.9 ± 2.05	8.72	7.54; 9.85	3.66–15.7
Tidal volume (mL)	292	399 ± 142	425	263; 500	110–714
End-tidal CO_2_ (kPa)	240	5.79 ± 0.82	5.7	5.21; 6.34	3.6–9.1
Compliance (mL/cmH_2_O)	20	43.7 ± 6.97	44	38.5; 48	30.8–55.8
**Arterial blood gas**					
pH	317	7.5 ± 0.06	7.49	7.45; 7.55	7.33–7.66
pO_2_ (kPa)	317	11 ± 1.63	11	10; 11.9	6.4–20.4
pCO_2_ (kPa)	318	5.82 ± 0.93	5.8	5.07; 6.5	3.6–8.78
aO_2_ (%)	317	96.1 ± 2.69	97	95.5; 98	78–100
BE (mmol/L)	317	9.32 ± 2.29	9.4	7.8; 10.9	0.35–17.4
Bicarbonate (mmol/l)	305	33 ± 3.07	33	31; 35	23-42.5
Lactate (mmol/L)	313	1.42 ± 0.53	1.3	1.1; 1.65	0.6–4.35
Na(mmol/L)	310	140 ± 2.69	140	138; 142	133–150
K (mmol/L)	318	4.25 ± 0.38	4.25	4; 4.5	3.1–5.45
iCa (mmol/L)	300	1.36 ± 0.07	1.36	1.32; 1.41	1.1–1.63
Glucose (mmol/L)	310	9.34 ± 2.36	9.3	7.68; 11.1	1.78–15.5
**Blood samples**					
Hemoglobin (g/L)	29	116 ± 10.1	116	113; 123	81–141
Platelets (10^9^/L)	51	362 ± 113	384	271; 447	111–631
WBC (10^9^/L)	29	10.7 ± 2.2	10.4	8.9; 12.5	5.9–14.7
Fibrinogen (g/L)	52	1.83 ± 2.35	1.45	1.2; 1.6	1-17.9
PK-INR	54	0.96 ± 0.06	1	0.9; 1	0.9–1.2
APT-T (s)	34	52.5 ± 34.5	56.5	9.23; 79.3	7-120
D-dimer (mg/L)	47	0.38 ± 0.67	0.25	0.2; 0.32	0.01–4.7
Prothrombin (kIE/L)	36	0.99 ± 0.13	0.98	0.9; 1.06	0.82–1.46
Protein C (kIE/L)	36	0.73 ± 0.15	0.75	0.69; 0.82	0.35–0.96
Troponin T (ng/L)	112	6.38 ± 9.85	5	0.41; 8.75	0.02-93
ALAT (µkat/L)	80	1.32 ± 0.27	1.29	1.14; 1.5	0.87–2.12
ASAT (µkat/L)	80	0.45 ± 0.44	0.4	0.33; 0.46	0.22–4.19
Creatinine (µmol/L)	29	110 ± 13.4	108	101; 119	85–140
LDH (µkat/L)	77	9.48 ± 1.68	9.55	8.65; 10.3	6.1–16.2
CK (µkat/L)	62	25.3 ± 26.5	16.5	10.2; 29.1	3.4–140

*Abbreviations* APTT = activated partial thromboplastin time; BE = base excess; CCO = continuous cardiac output; CK = creatine kinase; CVP = central venous pressure; LDH = lactate dehydrogenase; MAP = mean arterial pressure; MPAP = mean pulmonary arterial pressure; SAP = systolic arterial pressure; SD = standard deviation; SvO_2_ = venous oxygen saturation; WBC = white blood cells

### Arterial blood gas

Porcine blood has higher plasma buffer base concentration relative to man, and a greater capacity to compensate for acid loads [53]. We detected a higher pH than in man, and a mean base excess of 9.32. Swine pH neutrality differs at pH 7.5 and pCO_2_ 5.33 kPa which is attributed to a greater bicarbonate concentration of approximately 31 mmol/L compared to 24 mmol/l in man [53]. BE is defined as the amount of strong acid in mmol/L needed to restore pH neutrality (pH 7.4 at pCO_2_ 5.33 kPa and 37 °C in 1 L of human blood [54]. By this definition BE is zero mmol/L at pH neutrality in man. Thus, analyzing pH neutral porcine blood in a blood gas analyzer calibrated at human pH neutrality will result in a BE of around + 7–8 mmol/L instead of an actual BE of 0 mmol/l. Delta base excess is not constant through different pH levels, which is why swine specific nomograms may be required for correct BE [53]. Other skewed results may arise when analyzing porcine blood in blood gas analyzers calibrated for human blood, due to differences in baseline temperature, although we believe that the difference of 1 °C will not persist if a few minutes pass between the sampling and the analyzation.

### Anesthesia

Numerous acceptable anesthetic techniques for the swine exist, depending on the desired level of hypnosis, analgesia, and muscle relaxation. Techniques range from a single-drug injection, with minimal need for support and monitoring, to complex multiple drug protocols with invasive monitoring and intensive support [39]. In Table 3 and supplemental file 1, we report our standard protocol, which we have refined throughout the years to allow for severe trauma loads. We select ketamine because it is the drug of choice when anesthetizing hemorrhaging trauma patients, and thus clinically relevant. However, other medications may work equally well, depending on experimental protocol. Clinical anesthesiologists will note that the ketamine dose of 25 mg/kg/h is very high in comparison to man, which normally is 2–6 mg/kg/h for anesthesia maintenance. In our experience, this dose is necessary for adequate anesthesia. At 12.5 mg/kg/h the swine may respond to pain stimuli. The dose is in the range of previously described [2, 4, 39], and does not lead to severe distortions of baseline vital parameters.

**Table 3:** Anesthesia medications

	Mix in same syringe	
Premedication (IM)	Zoletil (tiletamine/zolazepam) (50 + 50 mg/mL) Dose 2.5 + 2.5 mg/kg	Medetomidine (1 mg/mL) dose 0.1 mg/kg
Induction (IV)	Pentobarbital (60 mg/mL) Dose: 6 mg/kg	
	Fentanyl (50 µg/mL) Dose: 2.5 µg/kg	
Maintenance (IV)	Ketamine (100 mg/mL) 25 mg/kg/h	Midazolam (1 mg/mL) 0.05 mg/kg/h
	Fentanyl (50 µg/mL) Dose: 3.5 µg/kg/h	

## Conclusion

60 kg swine share central anatomical and physiological features with man, which allows for seamless use of clinical monitoring equipment, medication, and physiological considerations.

## Electronic supplementary material

Below is the link to the electronic supplementary material.

Supplementary Material 1

## Data Availability

No datasets were generated or analysed during the current study.
