# Supplementary material for: The anesthesiologist’s guide to swine trauma physiology research: a report of two decades of experience from the experimental traumatology laboratory
Source: Eur J Trauma Emerg Surg. 2024 May 23;50(4):1879–89. doi: 10.1007/s00068-024-02542-7 (PMC11458652; doi:10.1007/s00068-024-02542-7)
Supplement: Supplementary file 1 — Supplementary Material 1 [file 68_2024_2542_MOESM1_ESM.pdf]

Supplemental material. Renberg et al. The anesthesiologist's guide to swine trauma physiology research: a report of two decades of experience from the Experimental Traumatology laboratory

<u>Premedication (intramuscular)</u>

Mixed in the same syringe:
Zoletil (dose 2.5 mg/kg) + Medetomidine/ (Cepetor) (dose 0.1 mg/kg)
Zoletil 50 mg/mL + 50 mg/mL. Cepetor 1mg/mL
Take an ampoule of Zoletil dry matter. mix with 10 mL of Medetomidine/Cepetor.
Withdraw 6 mL of that mixture.

<u>Induction (intravenous)</u>

Pentobarbital
Strength: 60 mg/mL
Dose: 6 mg/kg

$$\frac{\text{weight animal in kg x 6 mg/kg}}{\text{60 mg/mL}} = \text{volume in mL}$$

Fentanyl
Strength: 50 µg/mL
Dose: 2.5 µg/kg

$$\frac{\text{(Weight animal in kg x 2.5 µg/kg)}}{\text{50 µg/mL}} = \text{volume in mL}$$

Volume by animal weight

Weight (kg)	Thiopental (mL)	Fentanyl (mL)
55	5.5	2.75
56	5.6	2.8
57	5.7	2.9
58	5.8	2.9
59	5.9	3.0
60	6.0	3.0
61	6.1	3.1
62	6.2	3.1
63	6.3	3.2
64	6.4	3.2
65	6.5	3.3

<u>Maintenance (intravenous)</u>

Ringer-Acetate
3 mL/kg/h
Ketamine + Midazolam
Dose: Ketamine 25 mg/kg/h + Midazolam 0.0485 mg/kg/h

Mixed in 50cc Braun Omnifix Syringe:
33.5 mL Ketaminol 100 mg/mL + 6.5 mL Midazolam 1 mg/mL
Provides mixture with concentration 83.75 mg/mL Ketamine + 0.1625 mg/mL Midazolam.
Braun syringe pump: specify flow only (do not use medication list)

$$\frac{\text{Weight animal in kg x 25 mg/kg}}{\text{83.75 mg/mL}}$$ = mL/h in syringe pump

Fentanyl

Dose: 3.5 µg/kg/h
Inhaled without dilution in a 20 mL syringe.
Weight animal in kg x 3.5 µg = µg/h in syringe pump

$$\frac{\text{Weight animal in kg x 3.5 µg/kg/h}}{\text{50 µg/mL}}$$ = mL/h in syringe pump

Braun syringe pump: enter Fentanyl under medication List/Analgesics/Fentanyl

Volumes mL/h by animal weight in syringe pump

Weight (kg)	Ketamine (mL/h)	Fentanyl (µg/h)	Ringer-Acetate (mL/h)
55	16.4	192.5	165
56	16.7	196	168
57	17.0	199.5	171
58	17.3	203	174
59	17.6	206.5	177
60	17.9	210	180
61	18.2	213.5	183
62	18.5	217	186
63	18.8	220.5	189
64	19.1	224	192
65	19.4	227.5	195

Other medications

Noradrenaline
4 mL noradrenaline 1 mg/mL + 46 mL NaCl
Mixed in 50 mL Braun syringe.
Provides a strength mixture of 80 µg/mL.
Select protocol noradrenaline 80 µg/mL in Braun syringe pump.

Euthanasia
Pentobarbital sodium vet 100 mg/mL
35-40 mL (58.3 – 66.7 mg/kg for 60 kg animals)

Esmeron (Rokuronium)
Muscle relaxant
Strength: 10 mg/mL
Dose: 2 mg/kg
For 60 kg animals approx. 10 mL

Spontaneous Breathing Protocol

To allow spontaneous breathing to return as soon as possible after induction of the swine. this protocol must be started as soon as possible after induction. In case of randomization, it is mandatory that the swine has regained spontaneous breathing. Otherwise, this time is prolonged. Spontaneous breathing is defined in this context as $pCO_2 < 10$ kPa on the last blood gas before randomization + fulfilled stage 10 (completely self-triggered breathing).

1. After intubation, the following default settings are set. PCV+. PIP 15 cmH_2O. AF 20. PEEP 5 cmH_2O. PEEP 5 initially to counteract the inevitable development of atelectasis after induction. AF is adjusted to achieve normo-ventilation. $etCO_2$ approx. 5.3 kPa.
2. When the preoperative instrumentation is complete. the mode changes from PCV+ to PSIMV+ (you can also start with this setting). PEEP changes to 0 cmH_2O.
3. Lower the mandatory breathing rate from the initial position (AF approx. 18-22) to AF 15.
4. After another 15 minutes. the mandatory AF is lowered to 10.
5. After another 15 minutes. the mandatory AF is lowered to 5 (lowest adjustable value).
6. Before step 7. make sure the swine has started to trigger its own breathing. If not. remain on AF 5 for a while without initiating the next step to increase pCO_2.
7. After a further approx. 15 minutes. the trigger flow is reduced from 5 L/min to 2 L/min (lowest adjustable value).
8. After another 15 minutes. the PIP is reduced from 15 to 10 cmH_2O.
9. After another 15 minutes. the PIP is reduced from 10 to 5 cmH_2O.
10. After another 2-3 minutes. reduce to 3 cm H_2O. which corresponds to the tube compensation in the hose system.
11. Make sure the swine has pCO2 < 10 and triggers all the breaths itself.

Specific-pathogen-free (SPF) swine in Sweden are controlled for:

Pneumonia. Mycoplasma hyopneumoniae
Pleuritis. Actinobacillus Pleuropneumoniae (App)
Sneezing Disease. Atrophic Rhinitis
Transport sickness. Haemophilus parasuis
Dysentery. Brachyspira hyodysenteriae
Swine flu virus
Scabies
African swine fever virus
Aujeszky's disease virus
Japanese encephalitis virus
Foot-and-mouth disease virus
Porcine epidemiological diarrhea virus
PRRS virus
Rabies virus
Swine fever virus
Swine Vesicular Disease virus
Transmissible gastroenteritis virus
Brucella species
